# Supplementary material for: Vitamin D Modified DSS-Induced Colitis in Mice via STING Signaling Pathway
Source: Biology (Basel). 2025 Jun 18;14(6):715. doi: 10.3390/biology14060715 (PMC12190092; doi:10.3390/biology14060715)
Supplement: Supplementary file 1 [file biology-14-00715-s001.zip › Supplementary Materials S1: 16s-report of gut microbiota/Groups/diff_analysis/UnivarTestGenus/figure/barplot/alllog.pdf]

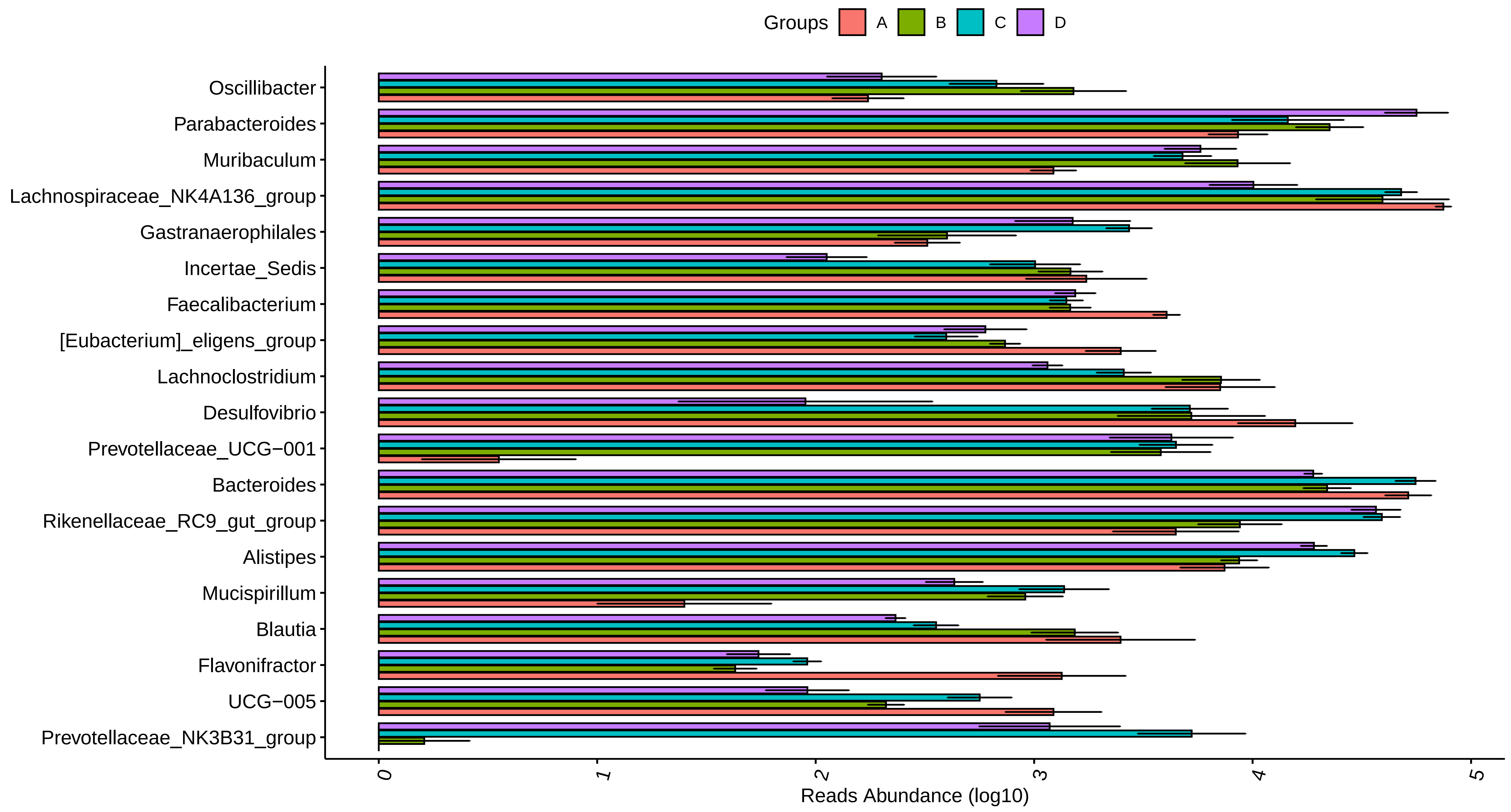
Groups
A
B
C
D
Oscillibacter
Parabacteroides
Muribaculum
Lachnospiraceae_NK4A136_group
Gastranaerophilales
Incertae_Sedis
Faecalibacterium
[Eubacterium]_eligens_group
Lachnoclostridium
Desulfovibrio
Prevotellaceae_UCG−001
Bacteroides
Rikenellaceae_RC9_gut_group
Alistipes
Mucispirillum
Blautia
Flavonifractor
UCG−005
Prevotellaceae_NK3B31_group
0
1
2
3
4
5
Reads Abundance (log10)
